# Defining Tumor Rupture in Gastrointestinal Stromal Tumor

**DOI:** 10.1245/s10434-019-07297-9

**Published:** 2019-03-13

**Authors:** Toshirou Nishida, Toto Hølmebakk, Chandrajit P. Raut, Piotr Rutkowski

**Affiliations:** 1 Department of Surgery, National Cancer Center Hospital, Chuoku, Tokyo Japan; 2 Department of Abdominal and Pediatric Surgery, Oslo University Hospital, The Norwegian Radium Hospital, Oslo, Norway; 3 Division of Surgical Oncology, Department of Surgery, Brigham and Women’s Hospital, Center for Sarcoma and Bone Oncology, Dana-Farber Cancer Institute, Harvard Medical School, Boston, MA USA; 4 Department of Soft Tissue/Bone Sarcoma and Melanoma, Maria Sklodowska-Curie Institute – Oncology Center, Warsaw, Poland

## Abstract

Tumor rupture is an important risk factor predictive of recurrence after macroscopically complete resection of gastrointestinal stromal tumors (GISTs), and an indication for defined interval or even lifelong adjuvant therapy with imatinib according to guidelines. However, there is no consensus or universally accepted definition of the term ‘tumor rupture’, and, consequently, its incidence varies greatly across reported series. Without predefined criteria, the clinical significance of rupture has also been difficult to assess on multivariate analysis of retrospective data. We reviewed the relevant literature and international guidelines, and, based on the Oslo criteria, proposed the following six definitions for ‘tumor rupture’: (1) tumor fracture or spillage; (2) blood-stained ascites; (3) gastrointestinal perforation at the tumor site; (4) microscopic infiltration of an adjacent organ; (5) intralesional dissection or piecemeal resection; or (6) incisional biopsy. Not all minor defects of tumor integrity should not be classified as rupture, i.e. mucosal defects or spillage contained within the gastrointestinal lumen, microscopic tumor penetration of the peritoneum or iatrogenic damage only to the peritoneal lining, uncomplicated transperitoneal needle biopsy, and R1 resection. This broad definition identifies GIST patients at particularly high risk of recurrence in population-based cohorts; however, its applicability in other sarcomas has not been investigated. As the proposed definition of tumor rupture in GIST has limited evidence based on the small number of patients with rupture in each retrospective study, we recommend validating the proposed definition of tumor rupture in GIST in prospective studies and considering it in clinical practice.

Sarcomas are a family of rare mesenchymal neoplasms consisting of over 100 pathologically and genetically heterogeneous tumors accounting for approximately 1% of all malignancies in adults. Fifteen percent are gastrointestinal stromal tumors (GISTs), 75% are non-GIST soft tissue sarcomas (STS), and 10% are osteogenic.^1^ GIST is the most common sarcoma of the gastrointestinal tract, with an estimated incidence of 1 per 100,000 per year.^1^ Most GISTs develop in the wall of the digestive tract or hollow viscera, and usually show expansive growth into the peritoneal cavity and/or gastrointestinal lumen. Although GIST is initially surrounded by normal gastrointestinal tissue, such as mucosa and serosa, breakdown of these biologic barriers, including the so-called pseudocapsule of compressed normal tissue, by tumor proliferation may result in spontaneous rupture, with subsequent dissemination of tumor cells into the peritoneal cavity. Tumor rupture may require emergent surgery and is usually associated with poor oncologic prognosis.

Although the goal of surgery for localized, resectable disease is a macroscopically complete resection, surgical manipulation with any incision into, or disruption of, the tumor capsule may result in potential dissemination of tumor cells into the peritoneal cavity. Extent of surgery has been described by various terms. The residual (R) tumor classification^2^ used in surgical oncology distinguishes macroscopic residual disease, microscopic residual disease at the surgical margins, and margin-negative resection, and applies to all solid tumors. The Enneking system,^3^ used almost exclusively in orthopedic oncology, distinguishes between a marginal and an intralesional dissection of soft tissue and bone sarcoma. The T4 category in the TNM system^4^ defines extra-compartmental growth into adjacent organs and tissues. In GIST, the term ‘tumor rupture’ is applied to the clinical scenario with both iatrogenic or spontaneous tumor exposure to the abdominal cavity or dissection field.

## The Concept of Tumor Rupture in Gastrointestinal Stromal Tumors (GIST) and Other Sarcoma

The prognosis of GIST patients depends on tumor size, mitotic count, and anatomic location. These anatomic and biological variables are included in the risk stratifications of the National Institutes of Health (NIH) consensus criteria,^5^ the Armed Forces Institute of Pathology (AFIP) classification,^6^ the Memorial–Sloan Kettering Cancer Center prognostic nomogram,^7^ and the Union for International Cancer Control/American Joint Committee on Cancer (UICC/AJCC) TNM classification.^4^ In addition to these anatomic and biologic factors, tumor rupture, a clinical factor, was introduced in the modified NIH risk classification based on a population-based study.^8^ The prognostic significance of tumor rupture was initially reported as an independent prognostic factor of gastrointestinal leiomyosarcomas,^9^ most of which would now be considered GISTs. Subsequently, tumor rupture was confirmed as a risk factor of recurrence in retrospective studies.^10^^–^19 Studies variably reported that tumor rupture was an independent prognostic factor predictive of worse recurrence-free survival (RFS),^10^,^12^^–^15 although this was not a consistent finding.^16^^–^19 Some studies demonstrated that recurrences after rupture were frequently peritoneal,^15^,^20^ whereas other studies did not confirm this.^13^,^14^ In any case, the risk of peritoneal or hepatic recurrence after tumor rupture is high, indicating that tumor rupture is an important prognostic factor in GIST.

Although relatively infrequent, tumor rupture in GIST may occur spontaneously before surgery or iatrogenically during surgical manipulation, and both etiologies are associated with similarly poor prognosis.^15^,^21^ Tumor rupture may be associated with biological aggressiveness, such as large tumor size, high mitotic count, and *KIT* exon 11 deletion mutations involving codons 557 and 558.^15^,^16^,^22^ Rupture may be relatively more frequent with small intestine GIST.^16^,^20^^–^22 There is no obvious association with use of neoadjuvant therapy or specific surgical approach (open vs. minimally invasive).^15^ In various clinical practice guidelines, adjuvant therapy is recommended for patients with ruptured GISTs.^1^,^23^,^24^ In contrast, spontaneous rupture is rarely reported for other STS, but iatrogenic rupture may increase the risk of local relapses and worsen disease-free survival (DFS). However, the objective or quantifiable clinical significance of tumor rupture remains undefined in non-GIST STS.

## Why Do We Need to More Precisely Define Tumor Rupture in GIST?

Although population-based data confirmed the negative prognostic impact of tumor rupture,^8^ the definition of rupture was not predefined a priori. With no established or consistent definition of tumor rupture, the reported incidence of tumor rupture varies greatly, from 1 to 27% (Table 1). In this context, it is not surprising that the independent impact on prognosis is not consistent on multivariate analysis.^12^^–^19 Some reports only included spontaneous rupture in their definition, while others also included iatrogenic rupture and even R1 surgery.^10^,^16^,^19^,^25^ These discrepancies have led to an inconsistent definition and, as a consequence, to an inconsistent prognostic impact of tumor rupture.^13^ Recently, a strict definition of tumor rupture was proposed by the Oslo Sarcoma Group.^13^,^14^,^22^ Their definition was comprised of six criteria and distinguished between tumor rupture and certain minor defects of tumor integrity not classified as rupture.^13^,^14^,^22^ In this review, we propose a comprehensive, evidence-based definition of what does and does not constitute tumor rupture in GIST for future validation and adoption.

**Table 1:** Studies reporting tumor rupture and related prognosis in GIST

References	Study characteristics	No. of patients	No. of patients with rupture (%)	Definition of rupture	Prognostic significance of rupture
Rutkowski et al.^10^	2002–2006; Polish clinical GIST registry; retrospective on rupture	335	75 (22)	Unspecified, R1 resection included	In multivariate analysis
Takahashi et al.^11^	1987–2003; Osaka region, Japan; retrospective	303	12 (4)	Unspecified	In univariate analysis
Rutkowski et al.^16^	2001–2010; Polish clinical GIST registry; retrospective on rupture	640	46 (7)	Spontaneous and intraoperative, otherwise unspecified	In univariate analysis only
Joensuu et al.^8^	1972–2010. Merged data from four European/Japanese studies; retrospective	1198	71 (6)	Unspecified	In multivariate analysis
Yanagimoto et al.^12^	1980–2010; Osaka region, Japan; retrospective	711	34 (5)	Unspecified, adjacent infiltration included	In multivariate analysis
Wozniac et al.^17^	1985–2012; ConticaGIST Registry, 13 European institutions; prospective	854	54 (6)	Unspecified	In univariate analysis only
Bischof et al.^18^	1998–2012; seven academic centers in US/Canada; retrospective	502	7 (1)	Unspecified	None
Casali et al.^25^	2004–2008; EORTC/Australasian trial (adjuvant imatinib); high/intermediate risk GIST	908	97 (11)	Unspecified, R1 resection included	None
Kim et al.^19^	2000–2007; South-Korean multicenter; gastric GIST; retrospective	1057	17 (2)	Intraoperative, otherwise unspecified	In univariate analysis only
Hølmebakk et al.^13^	2000–2012; South-East region, Norway; small intestinal GIST; retrospective	71	19 (27)	Spillage, fracture, incisional biopsy, bleeding, GI perforation, adjacent infiltration	In multivariate analysis
Hølmebakk et al.^14^	2000–2015; South-East region, Norway; gastric GIST; retrospective	242	22 (9)	Spillage, fracture, incisional biopsy, bleeding, GI perforation, adjacent infiltration	In multivariate analysis
Nishida et al.^15^	2003–2007; Kinki GIST Study Group, Japan; retrospective	665	21 (3)	Fracture and bleeding, otherwise individually defined	In multivariate analysis

*GIST* gastrointestinal stromal tumor, *EORTC* European Organization for Research and Treatment of Cancer, *GI* gastrointestinal, *R1 resection* microscopically positive resection margin

## The Proposed Definition of Tumor Rupture in GIST

**Fig. 1:**
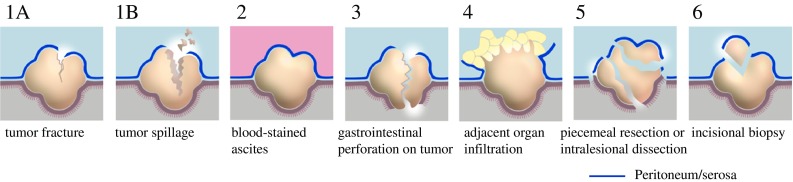
Cartoons illustrating tumor rupture in GIST. (**1a, 1b**) Tumor fracture and tumor spillage; (**2**) blood-stained ascites; (**3**) gastrointestinal perforation on tumor; (**4**) adjacent organ infiltration (microscopic); (**5**) piecemeal resection/intralesional dissection; (**6**) incisional biopsy. *GIST* gastrointestinal stromal tumor

We propose that six different clinical scenarios represent the spectrum of tumor rupture in GIST based on the Oslo criteria and supported by existing data (Table 2; Fig. 1). Furthermore, we list four additional clinical scenarios that would not be considered tumor rupture at this time based on the lack of supportive evidence (Table 2).

**Table 2:** Definition of tumor rupture in GIST

Conditions	Clinical settings	Suggestion
Tumor fracture and/or tumor spillage	Spontaneous or iatrogenic	Rupture
Blood-stained ascites	Spontaneous	Rupture
Gastrointestinal perforation through tumor	Spontaneous	Rupture
Microscopically direct tumor invasion into adjacent organs	Spontaneous	Rupture
Piecemeal resection or intralesional dissection	Iatrogenic	Rupture
Incisional biopsy	Iatrogenic	Rupture
Mucosal defect/intraluminal tumor perforation or gastrointestinal bleeding	Spontaneous	Non-rupture
Microscopic peritoneal penetration of tumor cells or iatrogenic peritoneal damage	Iatrogenic or spontaneous	Non-rupture
Core- or fine-needle biopsy without complications	Iatrogenic	Non-rupture
R1 resection	Iatrogenic	Non-rupture

*GIST* gastrointestinal stromal tumor, *R1 resection* microscopically positive resection margin

1. *Tumor Fracture and/or Tumor Spillage:* Tumor fracture may be spontaneous or iatrogenic. Spontaneous tumor fracture may require emergency surgery when associated with hemorrhage or peritonitis, whereas iatrogenic tumor fracture corresponds to an intralesional dissection according to the Enneking system.^3^ Iatrogenic tumor fracture or an intralesional dissection during surgery may result in poor prognostic outcomes due to potential dissemination of tumor cells, even if macroscopic complete resection was eventually performed. Tumor fracture has been also reported following trauma.^20^ In the SSGXVIII/AIO trial, tumor spillage at surgery was defined as intraoperative rupture.^26^
2. *Blood-Stained Ascites:* Blood-stained ascites may be evident at laparotomy and laparoscopy, and is sometimes associated with spontaneous tumor fracture, but macroscopic signs of rupture may be missing. In the SSGXVIII/AIO trial, this was defined as preoperative rupture.^26^
3. *Gastrointestinal Perforation at the Tumor Site:* Increase in luminal pressure, tumor fragility, or transmural tumor necrosis may result in spontaneous perforation with intraperitoneal tumor cell spillage (and spillage of intraluminal contents), a surgical emergency. This may be relatively rare but can happen to deeply ulcerated GISTs with thin tumor walls. Of note, a contained perforation within tumors (or tumor-enteric fistula)—as evidenced by gastrointestinal content or gas penetrating the tumor but without communication to the abdominal cavity on imagining, at surgery, or during pathology review—should not be considered rupture (Fig. 2).

**Fig. 2:**
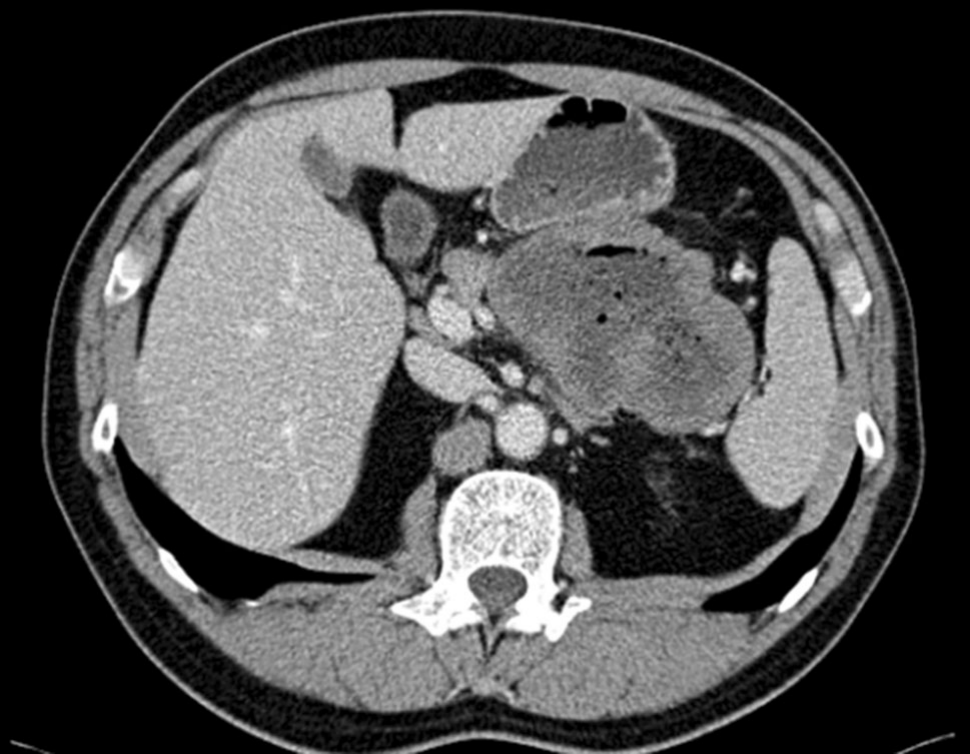
CT image of a contained perforation^1^ of a gastric GIST without communication to the abdominal cavity, which is not considered tumor rupture. ^1^A contained perforation; perforation into the gastrointestinal lumen without any communication to the abdominal cavity. *CT* computed tomography, *GIST* gastrointestinal stromal tumor
4. *Microscopic Adjacent Organ Infiltration:* This finding corresponds to T4b in the TNM classification of gastrointestinal carcinomas. We acknowledge that this may be a somewhat controversial category. Strictly speaking, it is not rupture, but represents longstanding peritoneal exposure of a biologically aggressive tumor, and, even if resected *en bloc* with clear margins, the prognosis is poor.^11^,^13^^–^15,27 In the Oslo series, all patients with this particular presentation relapsed despite R0 resections.^13^,^14^ Of note, fibrous or inflammatory adhesions without microscopic infiltration showed better prognostic outcomes and should not be considered rupture.
5. *Piecemeal Resection or Intralesional Dissection:* This is a variation of criterion 1 above (tumor fracture) and is invariably associated with spillage of tumor cells and extensive exposure of tumor tissue to the peritoneal cavity.
6. *Incisional Biopsy* This is usually a premeditated diagnostic procedure performed at laparotomy or laparoscopy, but, fortunately, this is a rare event in sarcoma centers. The evidence regarding this criterion is lacking. however it is technically a piecemeal resection. Thus, incisional biopsies should be discouraged in resectable primary GIST.

## Findings Not Considered Tumor Rupture

Some clinical settings with possible tumor exposure termed “minor defects of tumor integrity” by the Oslo Sarcoma Group^13^,^14^ show distinct outcomes from tumor rupture (Table 2). These clinical settings include (1) mucosal defects or tumor spillage into the gastrointestinal lumen with no extraluminal spillage; (2) microscopic tumor penetration of the peritoneum (which corresponds to T4a in the TNM classification of gastrointestinal carcinomas) or iatrogenic damage only to the peritoneal lining; (3) transperitoneal needle biopsy without complications; and (4) R1 resection (Table 2). The survival of patients with intact tumor resection is not different from that of patients undergoing surgery for tumors fulfilling one or several of these criteria, and patients in these two categories show better survival than those with tumor rupture as defined in the prior section; 5-year RFS rates in population-based series were 69% and 81% versus 36% in small intestinal GIST, and 96% and 91% versus 37% in gastric GIST, respectively.^13^,^14^

Of these, a potentially controversial category is peritoneal penetration. Iatrogenic damage to the peritoneal lining may be identified at surgery or histopathologic analysis. Under the microscope, it may be impossible to distinguish between an iatrogenic defect and a spontaneous penetration. These criteria were therefore grouped together in the studies investigating their prognostic significance;^13^,^14^ however, conceptually, they are different entities. When transperitoneal core needle biopsy is performed without complications, the procedure does not have an impact on prognosis.^28^ In GIST surgery, the resection margin corresponds to the organ transection surface or the extraperitoneal dissection surface—the circumferential margin. The peritoneum is without relevance to R status; hence, a GIST disrupted in terms of peritoneal penetration otherwise resected with negative margins is still considered an R0 resection. As demonstrated in the ACOSOG Z9000 and Z9001 trials,^29^ R1 resection is not an independent prognostic factor for recurrence on multivariate analysis. Mucosal defects (ulcer, endoscopic biopsy, piecemeal endoscopic resection), gastrointestinal bleeding, or tumor spillage into the gastrointestinal lumen are not classified as tumor rupture *per se*. However, tumor spillage within the peritoneal cavity during resection should be considered a potential peritoneal contamination, i.e. rupture.

The definitions of tumor rupture in GIST proposed in this work are based on the results of retrospective studies and experience from sarcoma centers, and have been shown to distinguish patients at high risk of recurrence in a restricted number of studies. However, these studies are all retrospective; the number of patients with tumor rupture are comparatively few, and the number within each category even fewer. The definitions need to be validated in prospective cohorts before widespread adoption. We elected to propose these definitions to collate the various categories of tumor rupture reported in the literature for further study.

## Management of Tumor Rupture

Tumor rupture is a strong and independent risk factor for recurrence, with long-term relapse rates of approximately 80%.^14^,^15^,^18^ Fifty percent of ruptures are spontaneous^15^ and are thus unpreventable, whereas iatrogenic rupture may potentially be avoided by referral to centers experienced in the multidisciplinary management of GIST. Rupture is related to tumor size, and neoadjuvant imatinib for 6–12 months should be considered for large tumors deemed at risk for rupture.^1^ Preoperative genotyping can also be considered in certain patients since gastric GISTs harboring a *KIT* exon 11 deletion involving codon 557 or 558 have increased risk of rupture (and could benefit from imatinib neoadjuvant treatment under appropriate circumstances).^22^ Rupture rates after neoadjuvant treatment are largely unknown, but studies have reported incidence rates ranging from 0 to 21%.^15^,^30^ Whether neoadjuvant treatment attenuates the dire consequences of rupture is unknown.

Tumor rupture is considered an indication for adjuvant imatinib therapy. However, most patients will relapse despite adjuvant treatment,^14^ and data from the SSGXVIII/AIO trial show that patients with rupture did not benefit from prolonged (3 years) adjuvant therapy.^31^ Extended adjuvant treatment is now being explored in two randomized trials (ClinicalTrials.gov identifier NCT02413736 and NCT02260505), but lifelong imatinib should be considered, as indicated in the European Society for Medical Oncology (ESMO) guidelines, essentially classifying these high-risk patients into a category similar to those with metastatic disease.^1^ Still, one in five patients will never experience a recurrence after rupture, and hopefully future research will enable clinicians to identify these patients who could be followed closely rather than treated.

## Clinical Significance of Tumor Rupture in Other Sarcomas

The clinical relevance of tumor rupture has been reported in STS.^32^ The quality of the initial surgery is an important factor for final outcomes for STS, and much research has confirmed that microscopically negative margins (R0) are a critical factor for local RFS and DFS.^33^^–^35 Local control has a significant impact on overall survival (OS) in specific localizations, such as retroperitoneal sarcomas (RPS).^36^,^37^ In extremity sarcomas, the relationship with OS is less evident, possibly due to salvage options such as reoperation, amputation, or regional infusion therapy in case of local relapse.^38^ Unplanned resection of a tumor and piecemeal resection in non-referral centers are associated with worse prognostic outcomes. Tumor rupture is extremely uncommon in extremity or trunk wall STS, and, in these localizations, tumor rupture is mainly iatrogenic as a result of inappropriate handling.^32^

Given location, RPS may be more prone to intraoperative rupture than extremity STS.^39^ Spontaneous rupture seems less common for RPS than for GIST.^40^,^41^ The long-term prognostic consequences of rupture for RPS, especially iatrogenic rupture, beyond local recurrence, are not as clear as for GIST. Perhaps, the most striking example of a sarcoma subgroup, besides GIST, in which tumor rupture has a negative impact on outcomes may be uterine sarcomas. The process of morcellation of a tumor during laparoscopic resection of undiagnosed uterine sarcoma definitively worsens the prognosis of patients, leading to inevitable dissemination of the sarcoma.^42^,^43^ Some studies suggest that patients having morcellation are most likely to develop distant rather than local recurrences, probably because tumor manipulation may cause disease spread into the upper abdomen and via hematological and lymphatic vessels.^44^

## Conclusions

Although tumor rupture is an important risk factor for recurrence after complete resection of GISTs for which adjuvant imatinib therapy is recommended according to guidelines, there has been no consistent definition of what constitutes ‘tumor rupture’ in GIST. Based on the Oslo criteria, we propose a comprehensive, composite definition of tumor rupture: (1) tumor fracture or spillage; (2) blood-stained ascites; (3) gastrointestinal perforation at the tumor site; (4) microscopic infiltration of an adjacent organ; (5) intralesional dissection or piecemeal resection; or (6) incisional biopsy. Minor defects, including mucosal defects or spillage contained within the gastrointestinal lumen, microscopic tumor penetration of the peritoneum or iatrogenic damage only to the peritoneal lining, uncomplicated transperitoneal needle biopsy, and R1 resection, should not be considered rupture. This definition is applicable for GIST, but not for non-GIST STS, where more evidence is required. Although some of the scenarios/categories are controversial, the proposed definition may identify GIST patients at particularly high risk of recurrence. We hope that this comprehensive definition of tumor rupture is validated in prospective studies.
